# Supplementary material for: Yiai Fuzheng decoction inhibits triple-negative breast cancer by remodeling the immune microenvironment
Source: Front Immunol. 2025 Sep 30;16:1615631. doi: 10.3389/fimmu.2025.1615631 (PMC12518410; doi:10.3389/fimmu.2025.1615631)
Supplement: Supplementary file 2 [file DataSheet1.zip › Supplementary File 2/Treatment/D_vs_H/07_Pathway_Analysis/Pathway_Analysis/Sig_Pathways_Name/Pyruvate metabolism.pdf]

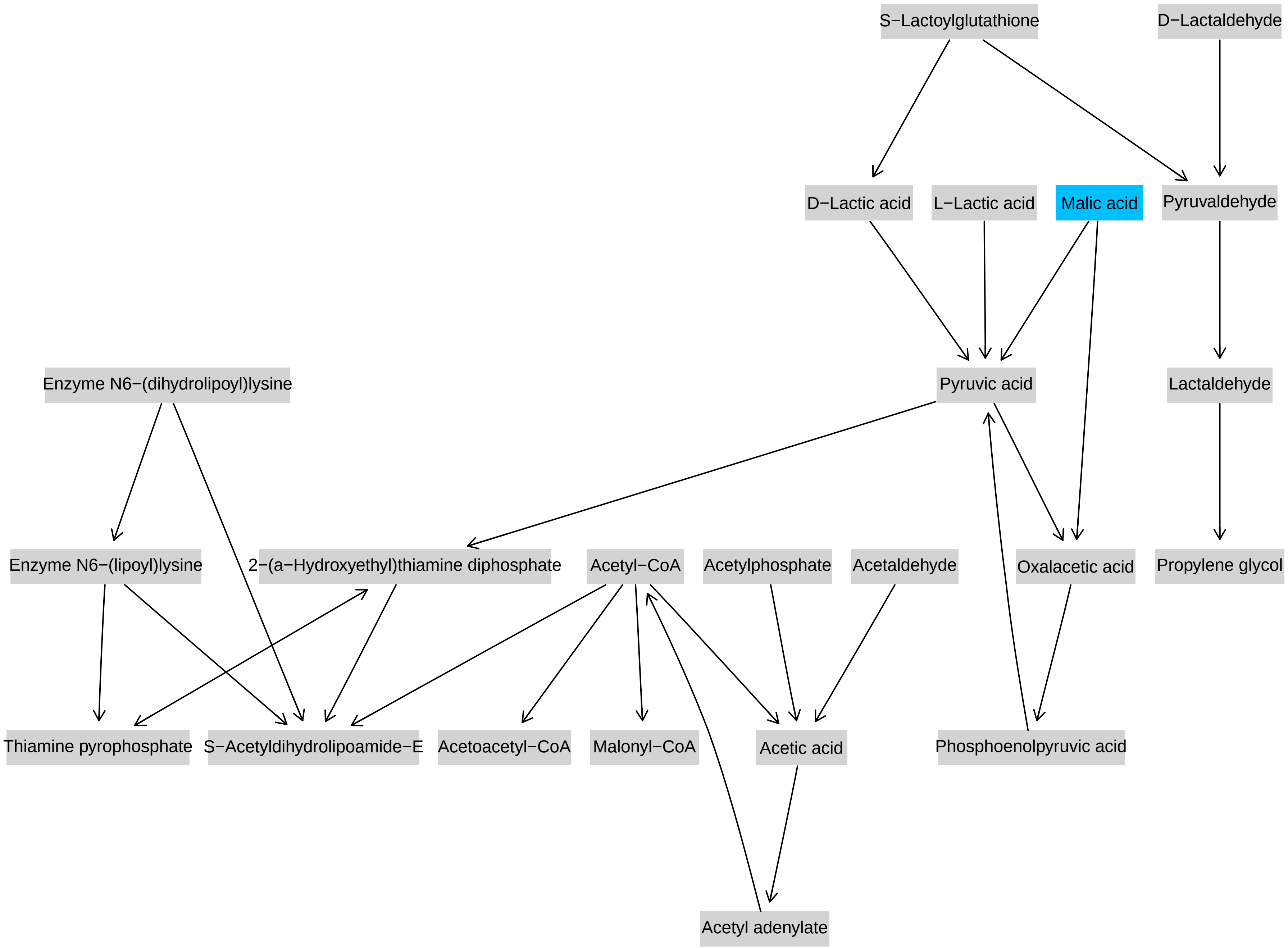
S−Lactoylglutathione
D−Lactaldehyde
D−Lactic acid
L−Lactic acid
Malic acid
Pyruvaldehyde
Enzyme N6−(dihydrolipoyl)lysine
Pyruvic acid
Lactaldehyde
Enzyme N6−(lipoyl)lysine
2−(a−Hydroxyethyl)thiamine diphosphate
Acetyl−CoA
Acetylphosphate
Acetaldehyde
Oxalacetic acid
Propylene glycol
Thiamine pyrophosphate
S−Acetyldihydrolipoamide−E
Acetoacetyl−CoA
Malonyl−CoA
Acetic acid
Phosphoenolpyruvic acid
Acetyl adenylate
